# Real world lessons on tyrosine kinase inhibitor response and resistance in CML from a developing country Pakistan

**DOI:** 10.12669/pjms.42.9.16564

**Published:** 2026-09

**Authors:** Sahrish Khan, Zafar Iqbal, Muhammad Usman Ghani, Muhammad Farooq Sabar

**Affiliations:** 1 Sahrish Khan, PhD, Center for Applied Molecular Biology (CAMB), University of the Punjab, Lahore, Pakistan; 2 Zafar Iqbal, PhD Department of Genomic & Experimental Medicine, King Saud bin Abdulaziz University for Health Sciences, Riyadh, Saudi Arabia; 3 Muhammad Usman Ghani, PhD, Center for Applied Molecular Biology (CAMB), University of the Punjab, Lahore, Pakistan; 4 Muhammad Farooq Sabar, PhD, Center for Applied Molecular Biology (CAMB), University of the Punjab, Lahore, Pakistan

**Keywords:** Imatinib resistance, Mutational profile, Nilotinib resistance, Real-world outcomes, Suboptimal molecular response

## Abstract

**Objectives:**

This study aimed to evaluate treatment responses, resistance and molecular characteristics of CML patients on TKI therapies to identify factors associated with suboptimal outcomes.

**Methodology:**

A retrospective observational study was conducted between January 2022 to December 2025 on 209 CML patients recruited at Mayo Hospital Lahore. Patients who achieved DMR and MMR were included. Minimum required duration of DMR was three years.

**Results:**

A total of 209 patients; 187 (89.4%) were in chronic phase, 12 (5.7%) in accelerated phase, and 10 (4.7%) in blast crisis. The majority were between 30–50 years of age and commonly observed splenomegaly (81.3%) and hepatomegaly (68.4%). Imatinib was prescribed to 114 patients (54.5%); however, 22 required a switch to nilotinib due to resistance.

Among CP-CML, 45 (24.0%) patients achieved deep molecular response (DMR) and 18 (9.6%) attained major molecular response (MMR), while one patient from accelerated phase exhibited MMR, leaving more than two-thirds with incomplete responses despite prolonged therapy.

Mutation analysis revealed G250E in a blast-phase patient with fatal outcome; another blast case with nilotinib resistance exhibited E459K and expired, E459K in accelerated-phase patient resistant to nilotinib. JAK2 (V617F) in CP-CML/MPN overlap two cases whereas 10 patients remained negative for JAK2 mutation. Death of five out of 10 blast patients have been confirmed.

**Conclusions:**

These real-world findings highlight the effectiveness of TKI therapy to achieving molecular responses, particularly in a substantial percentage of CP patients. However, variability in treatment response and emerging of resistance-associated mutations in advanced phases, emphasize the personalized management strategy.

## INTRODUCTION

Chronic myeloid leukemia (CML) is a myeloproliferative neoplasm characterized by the uncontrolled proliferation of myeloid cells in bone marrow and blood. CML commonly manifests with splenomegaly, hepatomegaly, thrombocytosis, thrombocytopenia, anemia, and left-shifted leukocytosis.^1^ The progression of CML occurs in three stages: chronic phase (CP), accelerated phase (AP) and blast crisis phase (BC), respectively. The classification of patients into these stages primarily depends on blast cell counts in the blood or bone marrow. Diagnostic techniques involved hematological, molecular, and cytogenetic assay which detect the blood cell count, quantification of BCR-ABL fusion gene and Philadelphia chromosome using blood and bone marrow.^2^ These techniques are essential for optimizing treatment, predicting prognosis, and improving patient survival.

Currently, three generations of TKIs are available: first generation (imatinib), second generation (bosutinib, dasatinib, and nilotinib), and third generation (ponatinib). These agents have markedly improved patient outcomes, allowing many to achieve life expectancies comparable to the general population.^3^ Worldwide, imatinib is recommended as the first-line therapy for CML.^4^ It effectively suppresses the proliferation of BCR-ABL1–expressing cells by blocking signaling pathways such as JAK-STAT and PI3K.^5^ Nilotinib is considered a protective and effective long-term option for patients who develop resistance to imatinib, showing both hematological and cytogenetic responses in most cases.^6^ With nearly 30-fold higher potency than imatinib, nilotinib can overcome imatinib resistance and is active against several kinase domain mutations, including F359V/I/C, Y253H, E255V/K, F317L/V/I/C, V299L and T315A, thereby offering improved therapeutic responses.^7^ TKI are potent therapeutic agents to suppress leukemic cell proliferation and induce durable molecular remission to significantly improve long term outcomes in patients. Early diagnosis is essential for the management of disease. Delayed evaluation may promote mutation and TKI resistance.^8^ Pakistan is among the low- and middle-income countries, where there are limited resources to manage the disease. The inability of advanced phase patients to achieve deep molecular responses despite prolonged therapy. Such evidence is essential to identify the factors associated with suboptimal therapeutic responses and highlights the need for earlier disease detection, treatment strategy and regular molecular monitoring to prevent the disease advancement and optimize clinical outcomes. This study aims to investigate therapeutic responses in CML patients and to address strategies for disease management to effectively reduce the burden of CML.

## METHODOLOGY

The retrospective observational study was conducted at the Oncology Department of Mayo Hospital, Lahore, Pakistan; from January 2022 to December 2025.

### Ethical approval:

The study was approved by the Ethical Committee of the hospital as well as by the University of the Punjab, Lahore (Ref. No. ID/115/FIMS, 10-10-2024).

Overall, 209 CML patients were recruited, of whom 106 (50.7%) were male and 103 (49.2%) were female. Written informed consent was obtained from all participants.

### Inclusion criteria:

The comprised clinically confirmed CML patients from the Pakistani population who were prescribed imatinib and nilotinib, covering all age groups and disease phases to capture a comprehensive spectrum. Patients who achieved DMR and MMR were included; the minimum required duration of DMR was three years. Patients who had complete clinical and laboratory records were enrolled.

### Exclusion criteria:

It included patients with diagnoses other than CML, those with secondary malignancies, and non-compliant individuals who missed follow-up for more than 12 months. Furthermore, All the hematology and molecular characteristics of patients were analyzed and documented at the interval of three-months. Therefore, demographic, CBC parameters, phases, scores, comorbidities and molecular profile along with treatment response was noted on data collection form. Based upon disease phases patients were classified into three groups CP, AP and BC phase. Risk scores (sokal, ELTS and EUTOS) were evaluated and monitored through MD+ calculator https://www.mdcalc.com and DEV calculator https://www.calculator.net/standard-deviation-calculator.html for suitable medication for CML patients.

### Statistical analysis:

Clinical and demographic characteristics were analyzed using descriptive statistics. Continuous variables were presented as mean values, whereas categorical variables were summarized as frequencies (n) and percentages (%).

## RESULTS

### Clinical profile of patients:

In the current study, a total of 209 CML patients were included and categorized into three phases of disease: CP-CML 187 (89.4%), AP-CML patients 12 (5.9%) and BC-CML included 10 (4.7%) patients. Overall, the cohort consisted of 106 males (50.7%) and 103 females (49.2%). The majority of patients in this cohort were within 30-50 years age group. CML patients commonly presented with bleeding 22 (10.9%), abdominal pain 48 (23.8%), vomiting 15 (7.4%), weight loss 46 (22.8%), sweating (0.9%), cough 13 (6.4%) and fatigue 30 (14.9%) as mentioned in Fig.1. Splenomegaly and hepatomegaly are one of the common factors in CML patients, occurring in 170 (81.3%) and 143 (68.4%) cases respectively. Across sub grouped patients, the splenomegaly was observed in 155 (82.8%) CP cases compared to 11 (91.6%) in AP-CML and 4 (40.0%) in BC-CML. Similarly, the prevalence of hepatomegaly was 133 (71.1%), 8 (66.6%), and 2 (20.0%) in CP, AP, and BC patients respectively.

**Fig.1:**
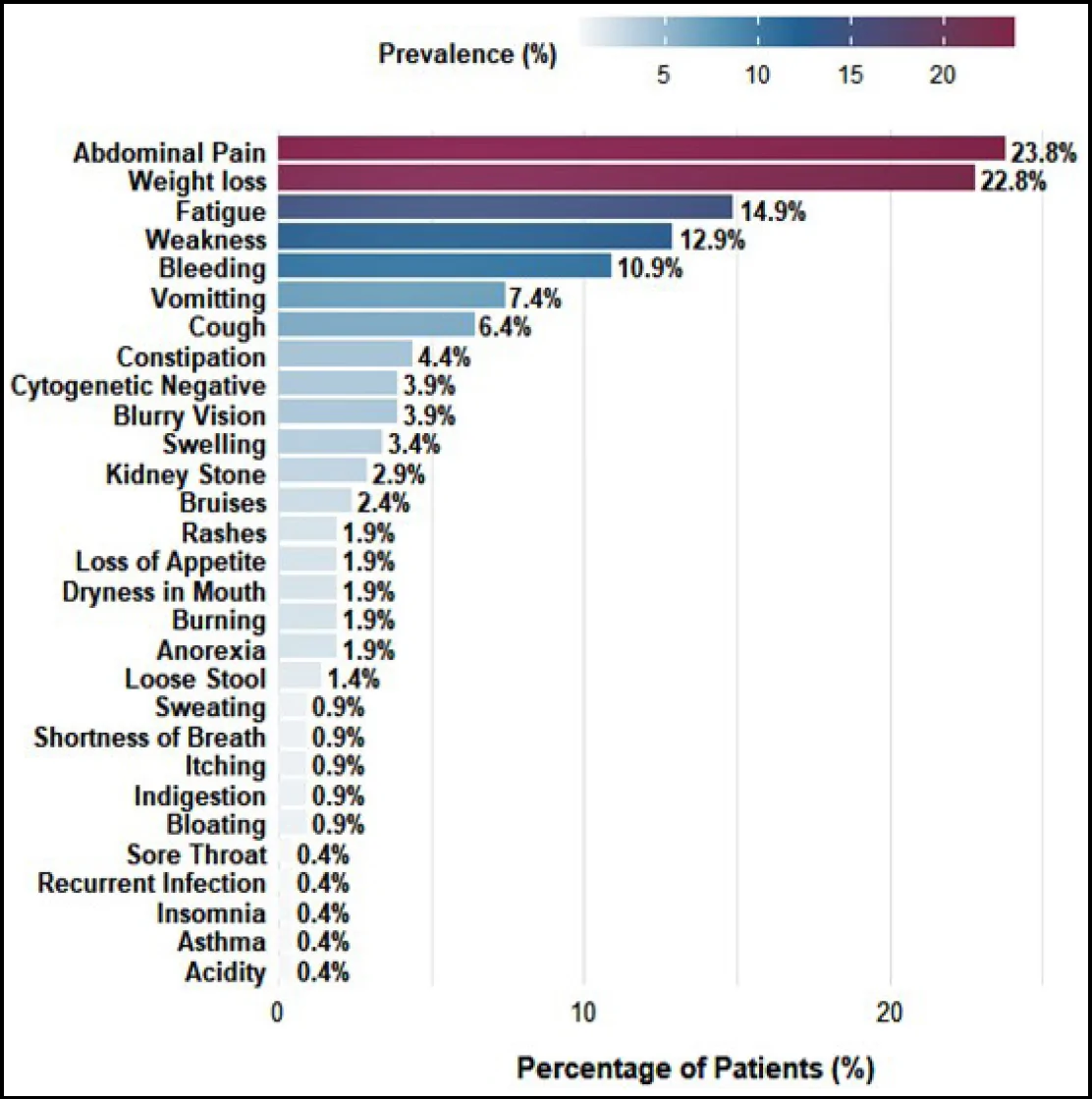
Prevalence of Clinical Symptoms in CML patients

### Risk scores:

Patients receiving imatinib 114 (54.5%) and nilotinib 95(45.4%) were evaluated by a prognostic scoring system. Among imatinib treated patients, 42 (37.5%), 5 (4.4%), and 77 (68.75%) had high sokal, EUTOS and ELTS scores respectively. Similarly, nilotinib treated patient revealed 56 (62.2%), 3 (3.3%) and 47 (52.8%) higher score of sokal, EUTOS and ELTS respectively. Nilotinib treated patients did not fall into low sokal scores. Sokal score 13 (11.6%) imatinib taking patients had low sokal score. The prognostic risk score of patients treated with imatinib and nilotinib were calculated as illustrated in Table-I.

**Table-I:** Risk Stratification of TKIs Treated Patients.

Imatinib N=114 (54.5%)	Sokal Score	Nilotinib N=95(45.4%)
13 (11.6%)	Low	0
56 (50%)	IM	33 (37%)
42 (37.5%)	High	56 (62.2%)
***EUTOS Score***		
45 (40.1%)	Low	49 (55%)
62 (55.3%)	IM	37 (41.5%)
5 (4.4%)	High	3 (3.3%)
***ELTS Score***		
4 (3.5%)	Low	2 (2.2%)
21 (18.75%)	IM	34 (38.2%)
77 (68.75%)	High	47 (52.8%)

*IM-Intermediate, Missing Data (N=7).

### Hematological and Clinical profile of study participants:

Anemia was present in 114 patients (54.5%), while leukocytosis (WBC >11×10^9^/L) was observed in 133 patients (63.6%). Thrombocytopenia (PLT <150×10^9^/L) occurred in 51 patients (24.4%). Only a few patients, 10 (4.7%) developed thrombocytosis (PLT >450×10^9^/L), whereas most showed improvement in platelet counts. Therefore, thrombocytosis and thrombocytopenia were common in CML individuals as mentioned in Table-II.

**Table-II:** Common Hematological Abnormalities in CML Patients.

Hematological Abnormalities	Values	Patients
Leukocytosis	WBC >11×10^9^/L	133 (63.6%)
Anemia	HB < 12g/dL	121 (57.8%)
Thrombocytopenia	PLT < 150×10^9^/L	51 (24.4%)
Thrombocytosis	PLT > 450×10^9^/L	10 (4.7%)
Splenomegaly	>12 cm	170 (81.3%)
Hepatomegaly	>15-16 cm	143 (68.4%)

Hematological abnormalities monitored among phases, all BC-CML patients had leukocytosis except one whereas 116 (62.0%) CP patients and 8 (66.6%) AP patients exhibited leukocytosis. More CP-CML patients 71 (37.9%) had a normal mean WBC count of 4-11×10^9^/L compared to other phases. All BC-CML patients were anemic with a standard of mean hemoglobin level < 12g/dL and in AP-CML, 10 (83.3%) majority of patients developed anemia. Among patients in CP-CML, more than 50% were anemic and 86 (45.9%) had normal hemoglobin level 12-16g/dL. 3 (30.0%) BC-CML patients had mean platelets counts within normal range 150-450×10^9^/L. Similarly, the high percentage of CP-CML 140 (74.8%) also maintained normal platelets counts. The mean PTLs count < 150×10^9^/L in 42 (22.4%) CP-CML and 3 (25%) in AP-CML whereas 6 (60.0%) patients with blast phase. Across all phases, some patients had an abnormal mean PLTs count exceeding > 450×10^9^/L as listed in Table-V.

**Table-III:** Demographic and Clinical Profile of CML Patients Characteristics by Phases.

Demographic/Clinical Information	Description	Phases		
	209 patients	CP-CML N=187 (89.4%)	AP-CML N=12 (5.7%)	BC-CML N=10 (4.7%)
Gender	106 (50.7%) Male	98 (52.4%) M	5 (41.6%) M	3(30.0%) M
	103 (49.2%) Female	89 (44.0%) F	7 (58.3%) F	7(70.0%) F
Age	≤10-30 years	60 (32.2%)	1 (8.3%)	1 (10.0%)
	30-50 years	86 (45.9%)	8 (66.6%)	7 (70.0%)
	50-70 years	41 (22.0%)	3 (25%)	2 (20.0%)
Blast cells	Range	1-10 %	11-20%	>20%
Splenomegaly	N=170	155 (82.8%)	11 (91.6%)	4 (40.0%)
Hepatomegaly	N=143	133 (71.1%)	8 (66.6%)	2 (20.0%)
Hematological Responses: White Blood Cell Count	Mean WBC 4-11×10^9^/L	71 (37.9%)	4 (33.3%)	1(10.0%)
	Mean WBC >11×10^9^/L	116 (62.0%)	8 (66.6%)	9 (90.0%)
Hemoglobin	Mean HB < 12g/dL	101 (54%)	10 (83.3%)	10 (100%)
	Mean HB 12-16g/dL	86 (45.9%)	2 (16.6%)	0
Platelets Count	Mean PLT < 150×10^9^/L	42 (22.4%)	3 (25%)	6 (60.0%)
	Mean PLT 150-450×10^9^/L	140 (74.8%)	5 (41.6%)	3 (30.0%)
	Mean PLT > 450×10^9^/L	5 (2.7%)	4 (33.3%)	1(10.0%)
Treatment	Imatinib	110 (58.8%)	2 (16.6%)	2 (20.0%)
	Nilotinib	77 (41.7%)	10 (83.3%)	8 (80.0%)
Molecular Responses: DMR	≤0.01%	`45 (24.0%)	0	0
MMR	≤0.1%	18 (9.6%)	1 (8.3%)	0

**Table-IV:** Compare BCR-ABL Quantification at Disease Diagnosis and time of Last Follow up in CML Patients.

Sr#	Initial BCR-ABL Quantification at Disease Diagnosis: No of Patients	Classified: Molecular Responses	BCR-ABL of Last Follow-up: No of Patients
1	0	DMR ≤0.01%	45
2	0	MMR ≤0.1%	19
3	22	>0.1%-10%	78
4	39	>10%-55%	34
5	144	>55%	12
6	2 (MD)	No BCR-ABL Test	14

* MD: Missing Data.

**Table-V:** ABL Kinase Domain Mutation of CML Patients.

Sr#	Mutation	ABL Domain	Technique	Phase	TKI Resistance	Survival
1	G250E	ABL1	Sanger Sequencing	BC-CML	Imatinib Resistance	Expired
2	E459K	ABL1	Sanger Sequencing	AP-CML	Nilotinib Resistance	Survive
3	E459K	ABL1	Sanger Sequencing	BC-CML	Imatinib, Nilotinib Resistance	Expired
4	V617F	JAK2	Sanger Sequencing	CP/CML-MPN	Imatinib Resistance	Survive
5	V617F	JAK2	Sanger Sequencing	CP/CML-MPN	Imatinib Resistance	Survive

### Treatment and Molecular response:

A total of 114 (54.5%) patients were treated with imatinib, 73 recommended for nilotinib based on initial diagnosis. Disease progressed and 22 patients from the imatinib treated group switched to nilotinib, exhibited imatinib resistance. Ninety five (45.4%) patients were in nilotinib treated group. However, nilotinib was frequently used in the advance phase patients as summarized in Table-III. BCR-ABL (p210) transcript was noted at the time of initial disease diagnosis and compared with last BCR-ABL, to evaluate the therapeutic responses as illustrated in Table-IV. During TKI treatment, 45 (24.0%) chronic patients achieved deep molecular response (DMR) and 18 (9.6%) CP-CML and only one patient from accelerated monitored with major molecular response (MMR). Molecular responses of 14 (6.9%) participants were not monitored for newly diagnosed patients; they had not yet completed their initial three months. At the time of disease diagnosis, 144 patients exhibited >55% BCR-ABL quantification whereas since the start of TKIs, only 12 patients revealed >55% BCR-ABL transcript as mentioned comparison in Table-IV. Some participants 8(3.9%) tested negative for Philadelphia chromosome while others were positive according to karyotyping analysis.

### Mutational profile:

The patient exhibited a mutation (c.749G>A:p.G250E), associated with imatinib resistance and nilotinib sensitivity, showed poor response with blast phase progression and subsequently expired. The second patient with nilotinib resistance harboring E459K variant and exhibiting abnormal hematological and molecular responses in AP-CML, was previously resistant to imatinib. A 45 years old female in blast phase could not respond well to TKIs. The disease progressed despite treatment, and patient developed resistance to imatinib and nilotinib. The patient harbored mutation E459K and exhibited anemia, leukocytosis and thrombocytosis, ultimately resulting in a fatal outcome. Among 10 blast patients, five expired in this study. Two patients were CML-MPN positive in chronic phase. All prognostic markers were at high risk, and the patient harbored the c.1849G>T, (p.V617F) mutation. The patient was resistant to imatinib but remained sensitive to nilotinib; therefore, nilotinib treatment was continued. Among the total, 10 (4.7%) patients were negative for JAK2 domain mutation.

## DISCUSSION

The mean age of the patients was 40 years, and the participants in the current study were within the age range of <10 to 70 years. This finding was consistent with a study conducted by Ngono et al., which reported a mean age of 39.2 years.^9^ Therefore, other studies reported a median age of 39 years and a range of 21-85 years.^10^ As our cohort included pediatric patients younger than 10 years, this indicates that CML also affects young individuals. Similar findings have been reported in other studies.^11^ Although the reason why younger individuals are affected by CML in developing countries remains unclear, a study from Switzerland reported that females accounted for 42.4% of cases, whereas males accounted for 57.6%.^12^ In our study, 106 males (50.7%) were affected compared to 103 females (49.2%). Thus, the proportion of males was slightly higher. Globally, both the incidence and mortality of CML are reported to be higher in males than in females.^13^ In India, where society is male-dominated, a higher incidence of CML has been reported among males, who also tend to receive more attention in society.^14^ Interestingly, females were more frequently affected in Saudi population. These findings highlight that epidemiological patterns may vary across different regions and populations.^15^ Disease severity is highest in BC, where 9 (90.0%) BC-CML patients in our study presented with leukocytosis. Leukocytosis was also observed in 116 (62.0%) patients in CP and 8 (66.6%) in AP. Only one BC-CML patient showed normal mean WBC counts (4–11 × 10^9^/L), and this group showed poor response to therapy with difficulty in achieving hematological remission. In contrast, CP is the initial and more manageable phase, with 71 (37.9%) patients maintaining normal mean WBC counts (4–11 × 10^9^/L). In a study from Turkey, Demircioğlu S et al., reported a median leukocyte count of 66.2 × 10^9^/L in 137 CML-CP patients.^16^ Similarly, a study from Quetta including 131 CML patients reported leukocytosis in 75 (57.2%) cases with WBC counts >100 × 10³/μl.^17^ In our cohort, leukocytosis was noted in 133 patients (63.6%). These findings contrast with our results, particularly in the number of patients in chronic phase. Among the overall study population, thrombocytopenia was detected in 51 patients (24.4%) and thrombocytosis in 10 patients (4.7%). However, our results differ from those who described a CML case presenting with marked thrombocytosis exceeding 2000 × 10^9^/L.^18^ In our study, these factors were not analyzed in the blast phase because of small sample size. In our hospital, the most frequently used risk stratification tool was sokal score. In our cohort, 56 (62.2%) nilotinib-treated patients had high scores compared with 42 (37.5%) in the imatinib-treated group. Notably, none of the patients in the nilotinib group fell into the low sokal score category, which may be attributed to abnormal blood parameters contributing to higher scores. This finding contrasts with a previous report in which 47% of nilotinib-treated patients had the highest sokal scores.^19^ Our results were consistent with studies conducted in China and India. In our cohort, a larger proportion of patients 114 (54.5%) initially received imatinib; however, as the disease progressed, 22 patients developed imatinib resistance and were switched to nilotinib. Imatinib resistance is commonly associated with mutations such as G250, Y253, E255, V299, and T315.^20^ In our study, 45 (24.0%) CP patients achieved DMR, and attained 18 (9.6%) MMR while only one patient in AP achieved MMR at the time of presentation. These findings differ from those of Iurlo A et al., who reported that 48% of patients achieved DMR 84.1% achieved DMR and treatment-free remission (TFR), while 71.1% attained MMR during TFR.^21^

One nilotinib resistance AP-CML patient had variant p.E459K in ABL domain, while another blast phase patient exhibited mutation E459K, resistant to both imatinib and nilotinib and expired in our cohort. These findings were similar to Seman ZA et al., who reported imatinib resistant accelerated phase patient had this pathogenic variant in Malaysian population.^22^ However, compare with our study and revealed differences in phases, same mutation, whereas patients received different TKIs, reflecting differences between Pakistani and Malaysian participants, which might be based on treatment availability, clinical guidance and genomic complexity. In the current study, c.749G>A (p.G250E) mutation was detected in a blast-phase patient with imatinib resistance and ultimately expired. Among 10 blast patients, five expired in our study. Similarly, a study from Malaysia also reported the G250E mutation in imatinib-resistant patients.^23^ Another study conducted from Cote ˆ d’Ivoire and reported 5 deaths among 72 patients; these participants had mutations such as, three patients exhibiting T3151, including one with compound mutation T3151/M244V. All these patients were treated with imatinib and revealed compound mutations G255V and H396P/E459K.^24^ However, the emergence of multiple ABL mutations required standardized NGS based smart mutation screening tools for clinicians to manage TKIs resistance. Therefore, CML real world findings demonstrated the effectiveness of TKI therapy in achieving molecular responses, particularly in patients with chronic-phase CML; 45 patients achieved DMR and 19 achieved MMR. However, variable treatment responses and resistance-associated mutations, particularly in advanced disease phases, highlight the need for personalized treatment and regular molecular monitoring.

### Strengths

The strength of our study lies in its real-world data and longitudinal assessment of molecular responses and mutations.

### Limitations

Pakistan faces significant challenges in managing disease due to a lack of advanced molecular techniques to monitor disease and limited cancer care hospitals. 2^nd^ generation (except nilotinib) and 3rd generation TKIs are not available from government health departments.

## CONCLUSIONS

Age is a risk factor for leukemia and a 30–50 years group is more affected with CML. Most CML patients presented with leukocytosis, thrombocytopenia, anemia, splenomegaly and hepatomegaly. Being a heterogeneous disorder, resistance has become a common factor. However, the emerging mutations in BCR-ABL and non-BCR-ABL increase the disease burden and diminish therapeutic responses. Resistance highlights the demand for further validation of patient characterization that may open new avenue new monitoring approaches and personalized therapy to improve patient outcomes in cancer settings.

## Recommendations

Further multicenter studies with larger cohorts and longer follow-up are needed to identify predictive biomarkers and optimize personalized, mutation-guided treatment strategies.
